# Miller-Fisher Syndrome Unveiled in the Presence of Cholangiocarcinoma

**DOI:** 10.7759/cureus.49016

**Published:** 2023-11-18

**Authors:** Narek Hakobyan, Ruchi Yadav, Akriti Pokhrel, Mustafa Wasifuddin, Michaela J John, Siddharth Yadav, Avezbakiyev Boris

**Affiliations:** 1 Internal Medicine, Brookdale University Hospital and Medical Center, Brooklyn, USA; 2 Hematology and Oncology, Brookdale University Hospital and Medical Center, Brooklyn, USA; 3 Internal Medicine, St. George's University School of Medicine, Brooklyn, USA; 4 School of Health Sciences, The Bronx High School of Science, Bronx, USA

**Keywords:** bickerstaff brainstem encephalitis, guillain-barré syndreome, distal cholangiocarcinoma, intrahepatic cholangiocarcinoma, miller-fisher syndrome

## Abstract

Miller-Fisher syndrome (MFS) is a rare variant of Guillain-Barré syndrome, characterized by ataxia, areflexia, ophthalmoplegia, and possible facial, swallowing and limb weakness alongside respiratory failure. Variations within MFS may include respiratory and limb weakness and Bickerstaff brainstem encephalitis (BBE), marked by altered consciousness, ataxia, ophthalmoparesis, and paradoxical hyperreflexia. MFS can emerge in both children and adults, often following bacterial or viral illness. While autoimmune-driven nerve damage occurs, most MFS patients recover within six months without specific treatment, with a low risk of lasting neurological deficits or relapses. Rarely fatal, MFS’s co-occurrence with cholangiocarcinoma (CCA) presents unique management challenges. CCA, primarily affecting bile ducts, has a bleak prognosis; surgical resection offers limited cure potential due to late-stage detection and high recurrence rates. Advances in CCA’s molecular understanding have led to novel diagnostic and therapeutic approaches, requiring a comprehensive interdisciplinary care approach for optimal MFS and CCA management outcomes.

Herein, we present a 50-year-old male with a complex medical history who was admitted to the hospital due to abdominal discomfort, nausea, vomiting, and ascites. Imaging revealed pneumonia and secondary bacterial peritonitis. Later, he developed neurological symptoms, including weakness, gait abnormalities, and brainstem symptoms, leading to the diagnosis of MFS. Despite treatment efforts, his condition deteriorated, leading to acute liver failure and unexplained anasarca. N-acetyl cysteine was initiated for liver issues. Neurologically, he showed quadriparesis and areflexia. Intravenous immunoglobulin (IVIG) treatment improved his neurological symptoms but worsened gastrointestinal issues, including ileus and elevated CA19-9 levels, suggesting a potential carcinoma. A liver biopsy was performed. After IVIG treatment, he experienced widespread discomfort, emotional unresponsiveness, swallowing difficulties, and aspiration risk, ultimately leading to his demise.

## Introduction

Miller-Fisher syndrome (MFS) constitutes a rare variant within the spectrum of Guillain-Barré Syndrome (GBS), distinguished by a distinctive triad involving ophthalmoplegia, ataxia, and areflexia. Notably, it frequently manifests in association with antecedent infections, most notably *Campylobacter jejuni* infection [1]. However, the concurrent manifestation of MFS alongside an underlying malignancy, such as cholangiocarcinoma (CCA), represents an exceedingly infrequent event and has garnered limited scrutiny within the corpus of medical literature. CCA, a malignant neoplasm arising from the biliary epithelial cells, assumes heterogeneous clinical manifestations contingent upon its anatomical location and developmental stage [2]. While instances of neurological paraneoplastic syndromes have been documented concomitantly with diverse malignancies, the concurrent emergence of MFS and CCA presents an exceptional and captivating phenomenon warranting further comprehensive exploration.

Within this context, we present a distinctive clinical vignette featuring a patient diagnosed with CCA, subsequently exhibiting clinical indicators consonant with MFS. A meticulous exposition of the patient’s clinical trajectory is furnished, encompassing the intricacies of diagnostic intricacies encountered and the therapeutic modalities deployed to address this singular concurrence. Our illustrative account underscores the compelling imperative for heightened vigilance and exhaustive assessment in instances where patients diagnosed with CCA concurrently display neurological symptomatology evocative of MFS. Furthermore, our endeavor seeks to bolster cognizance among the cadre of healthcare practitioners while concurrently engendering impetus for further scholarly investigation into the underlying pathophysiological substrates governing the co-occurrence of these intricate maladies.

## Case presentation

**Figure 1:**
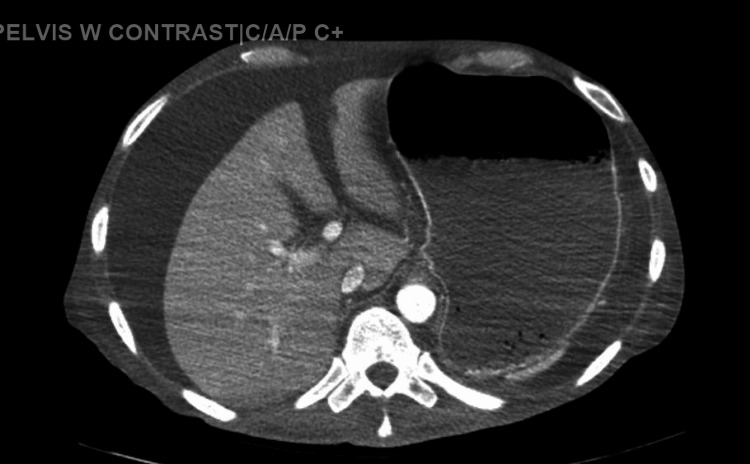
CT image showing ascites fluid around the liver with an enlarged stomach

The subject under examination is a male individual of 50 years who bears a medical history that encompasses type 1 diabetes mellitus, utilization of opioids, resolved hepatitis C infection, and Crohn’s disease. The patient sought medical attention at the hospital following the manifestation of abdominal discomfort accompanied by nausea in conjunction with two incidents of emesis transpiring over a span of 24 hours. The preliminary physical assessment unveiled the presence of abdominal tenderness and guarding devoid of focal localization alongside the elicitation of a positive fluid wave sign. Subsequent computed tomography imaging disclosed the extensive occurrence of ascites alongside bilateral pneumonia, thereby culminating in the administration of ceftriaxone, azithromycin, and metronidazole. The CT image is shown in Figure 1.

Over a subsequent interval of two days, the patient experienced repetitive bouts of emesis characterized by a coffee-ground appearance, accompanied by a conspicuous escalation in abdominal distension and an ongoing exacerbation of abdominal pain. A bedside paracentesis procedure was undertaken, leading to the withdrawal of 2 L of serous/cloudy ascitic fluid. Furthermore, due to an elevated international normalized ratio of 2.56 and prothrombin time of 29.0 seconds (reference range: 10-13 seconds), the patient received a transfusion of 2 units of fresh frozen plasma. The initial admission labs are shown in Table 1. 

**Table 1:** Complete blood count, renal function tests, and coagulation panel on the day of admission

Lab	Value	Reference
WBC	16.2	4.1-10.1 10x3 /uL
HGB	11.3	12.9-16.7 g/dL
HCT	32.7	40-47%
PLT	475	153-328 10x3 /uL
BUN	35.8	9.0-20.0 mg/dL
Cr	0.46	0.66-1.25 mg/dL
PTT	39.9	23.5-35.5 sec
PT	19.4	9.2-12.8 sec
INR	1.62	0.70-1.20

Paracentesis findings revealed peritoneal fluid of anomalous coloration and cloudy appearance, exhibiting elevated cellular counts, including 23,250 cells/uL of WBCs (high) predominantly comprised of neutrophils (91%) and 4,500 cells/uL of RBCs (high). Aerobic culture identified clustered gram-positive cocci with methicillin-sensitive *Staphylococcus aureus*, affirming secondary bacterial peritonitis as the diagnosis.

Subsequent to a five-day interval from admission, the patient underwent physical therapy assessment, unveiling a constellation of symptoms, including generalized weakness, functional deterioration, gait abnormalities, and compromised upper extremity strength (3/5) accompanied by reduced effort and resistance. Further neurological evaluation documented coexistent neuropathic and upper motor neuron signs, coupled with upbeat nystagmus upon upward gaze, indicative of brainstem involvement. A lumbar puncture yielded a negative WBC count but revealed elevated protein levels (89 mg/100 mL; reference range: 15-60 mg/100 mL).

Despite therapeutic efforts, the patient’s condition deteriorated, necessitating transfer to the medical intensive care unit due to acute liver failure. Concurrently, the patient displayed a cachexic appearance and distended abdomen, with unexplained anasarca despite inconclusive findings on ultrasound and echocardiography. Initiating a course of N-acetyl cysteine protocol aimed at addressing elevated liver enzymes and suspected nonalcoholic fatty liver disease proved vital. Neurologically, facial muscle power remained intact, along with full extraocular movement and the presence of upbeat nystagmus on upward gaze. The patient exhibited quadriparesis and areflexic traits, with diminished pain sensation below the upper shins yet retained proprioception in digits.

Pending antibody screening outcomes, the patient’s care transitioned to the telemetry unit, where intravenous immunoglobulin (IVIG) treatment was initiated. Encouragingly, the initial doses of IVIG led to mild amelioration in quadriparesis. However, concurrent with these neurological gains, gastrointestinal symptoms intensified, evidenced by gastric and transverse colon distension, coupled with fecal retention observed in imaging. The prompt intervention included NPO status and Salem sump insertion for gastric decompression. A follow-up abdominal X-ray disclosed an escalating ileus. Serum analysis unveiled an elevated CA19-9 level of 232, implying a potential carcinoma etiology. Furthermore, given the persistently deranged liver enzymes, interventional radiology was engaged for a liver biopsy. Laboratory findings post-initiation of IVIG treatment are shown in Table 2.

**Table 2:** Complete blood count, renal function tests, and coagulation panel on day 22 of admission, post-IVIG IVIG, intravenous immunoglobulin

Lab	Value	Reference
WBC	4.8	4.1-10.1 10x3 /uL
HGB	8.3	12.9-16.7 g/dL
HCT	24.7	40-47 %
PLT	31	153-328 10x3 /uL
BUN	66.0	9.0-20.0 mg/dL
Cr	0.36	0.66-1.25 mg/dL
PTT	30.2	23.5-35.5 sec
INR	1.55	0.70-1.20
ALT	551	<50
AST	>1,500	17-59 U/L
Alk Phos	>3,000	38.0-126.0 U/L

Following the administration of a regimen comprising five doses of IVIG, the patient conveyed a sensation of widespread and diffuse bodily discomfort. Concurrently, his emotional demeanor exhibited a lack of affective responsiveness, prompting consultation with the palliative care team. Compounding the clinical picture, the patient encountered difficulties in swallowing, necessitating a comprehensive assessment. A test protocol involved the administration of a singular half teaspoon of pureed applesauce and a small volume of water. Notably, this elicited distinct wet gurgling phonation and upper airway sonorities coincident with water ingestion. As a consequence of the perceived risk of aspiration, the decision was made to enforce NPO status. Regrettably, the observed paralysis demonstrated no propensity for amelioration, culminating in the patient’s eventual succumbing to his affliction.

## Discussion

MFS represents a relatively uncommon subtype of GBS characterized by a specific triad of clinical manifestations, encompassing ataxia, areflexia, and ophthalmoplegia [1]. However, the clinical presentation of MFS may encompass additional neurological indicators. There is an established predilection for male patients in terms of infectivity rates [3]. The collective spectrum of GBS, including its various variants, materializes at an average age of 43.6 years, with the incidence displaying a positive correlation with advancing age [4].

MFS is frequently associated with an antecedent upper respiratory tract or gastrointestinal infection, although autoimmune or neoplastic etiologies may also contribute to its onset [5]. Notably, inflammatory bowel conditions such as Crohn’s disease, an autoimmune disorder, have been linked to both GBS and its Miller-Fisher variant [6]. Our patient, during the course of hospitalization, was found to possess Crohn’s disease with anti-ASCA positive antibodies, which bear a sensitivity of 80%. Remarkably, the coexistence of MFS with CCA, an infrequent neoplastic ailment, remains a distinct clinical scenario with scant precedent within the medical literature. Despite initial indications of gastrointestinal infection, subsequent investigations revealed an underlying CCA as the instigator of the patient’s symptoms.

In severe cases, the ataxic component of MFS can precipitate a loss of ambulatory ability [7]. Our patient exhibited early ataxia in the clinical course, culminating in a fall resulting in chin bruising while attempting ambulation. MFS’s symptomatology can overlap with GBS, potentially extending to quadriparesis and respiratory insufficiency [8]. Differential diagnostic considerations when assessing MFS include vascular pathologies involving the brainstem, botulism, and myasthenia gravis, necessitating a comprehensive exclusionary approach [9]. The diagnostic methodology for MFS generally relies on clinical history, with a noteworthy majority of patients describing prior symptomatic infectious encounters [10]. Lumbar puncture is often employed to support neurologic diagnoses, with a distinctive profile of normal cell counts paired with heightened protein levels in cerebrospinal fluid classic for MFS [11]. In our patient’s case, cerebrospinal fluid analysis revealed glucose and protein values of 98 mg/100 mL and 89 mg/100 mL, respectively, consistent with MFS. Serological confirmation of MFS is attainable through the detection of anti-GQ1b antibody titers, exhibiting a high specificity and a positive correlation with ophthalmoplegia severity [7].

MFS typically follows a self-limiting course; however, evidence indicates the potential benefit of immunotherapeutic interventions [5]. Notably, no randomized controlled trials have comprehensively evaluated treatment modalities for MFS. IVIG has been shown to expedite recovery to the remission phase, albeit with limited impact on long-term outcomes [7]. According to Rath et al., a recommended adult dose of IVIG is 2 g/kg of body weight administered over five days [12]. Consequently, our patient received an intravenous dose of 400 mg/kg of IVIG over a span of five days. Typically, symptomatic improvement becomes evident within two to four weeks post-IVIG initiation, with complete symptom resolution observed within six months from symptom onset [9]. The effective management of pain stands as a pivotal determinant influencing hospital recovery. As motor function declined, aggressive medical management was undertaken. The removal of a nasogastric tube, while addressing pain, inadvertently resulted in compromised nutrition, further retarding recovery. A proactive approach to pain management involving a combination of analgesic agents significantly impacts the pace of recuperation [9]. Similarly, early engagement with physical therapy stands as a fundamental constituent in the management and convalescence of MFS [9].

CCA presents diagnostic challenges, characterized by its latent clinical course and a lack of definitive diagnostic criteria [13]. The serum biomarker cancer antigen 19-9 (CA 19-9) is employed prominently for CCA diagnosis, with CA 19-9 levels exceeding 1,000 U/mL associated with metastatic involvement [14]. Our patient demonstrated a CA 19-9 level of 232 U/mL, suggesting a limited likelihood of metastatic dissemination. Histopathological assessment of biopsy specimens substantiates CCA diagnosis [14]. Clinical presentation often occurs at an advanced stage, portending an unfavorable prognosis [13]. While de novo development predominates, risk factors including diabetes, tobacco use, nonalcoholic fatty liver disease, nonalcoholic steatohepatitis, and hepatitis B or C infections are significantly associated with CCA risk [13,15]. Surgical intervention stands as the singular curative option; however, a substantial subset of advanced-stage patients are ineligible for surgery or have unresectable disease [14]. Cumulatively, given the grim prognosis, patient mortality mirrors incidence rates [14].

## Conclusions

Comprehending the intricate interplay between MFS and CCA holds substantial significance, owing to its potential ramifications encompassing diagnostic refinement, therapeutic paradigms, and prognostic determinants. The acknowledgment of a cohesive link between these entities stands to expedite the timely detection of latent neoplastic conditions within individuals manifesting atypical neurological manifestations, thus affording opportune interventions and augmented clinical outcomes. Heightened cognizance coupled with ongoing scholarly inquiry into this intriguing nexus promises to unravel deeper insights into the fundamental pathophysiological underpinnings, thereby culminating in the refinement of tailored management approaches for this unique patient cohort.
